# Prevalence of chronic obstructive pulmonary disease (COPD) in China in 1990 and 2010

**DOI:** 10.7189/jogh.07.020704

**Published:** 2017-12

**Authors:** Kit Yee Chan, Xue Li, Wanjing Chen, Peige Song, Nuen Wing Katy Wong, Adrienne N Poon, Weiyan Jian, Ireneous N Soyiri, Simon Cousens, Davies Adeloye, Aziz Sheikh, Harry Campbell, Igor Rudan, on behalf of Global Health Epidemiology Research Group (GHERG)

**Affiliations:** 1 Centre for Global Health Research, Usher Institute for Population Health Sciences and Informatics, The University of Edinburgh, Scontland, UK; 2 Nossal Institute for Global Health, University of Melbourne, Melbourne, Australia; 3 Sichuan Academy of Medical Sciences, Chengdu, China; 4 Sichuan Provincial People's Hospital, Chengdu, China; 5 Hospital Authority, Hong Kong, China; 6 Department of Internal Medicine, School of Medicine & Health Sciences, The George Washington University, Washington, D.C., USA; 7 Department of Health Policy and Management, School of Public Health, Peking University Health Science Centre, Beijing, China; 8 Centre for Medical Informatics, Usher Institute for Population Health Sciences and Informatics, The University of Edinburgh, Scotland, UK; 9 Department of Infectious Disease Epidemiology, London School of Hygiene and Tropical Medicine, Keppel Street, London, UK; 10 Demography and Social Statistics, and the e–Health Research Cluster, Covenant Universit, Ota, Ogun State, Nigeria; * Joint–first authors

## Abstract

**Background:**

Chronic obstructive pulmonary disease (COPD) is set to become the third most frequent cause of death and also the third largest cause of global morbidity by 2020. In China, where the population is aging rapidly, COPD has become one of the leading causes of disability and a large economic burden. An epidemiological assessment of the COPD in China is required, with a focus on the number of cases living with disease, main determinants of the disease and time trends.

**Methods:**

We systematically searched large Chinese bibliographic databases and English databases to identify spirometry–based epidemiological studies of the prevalence of COPD in China diagnosed according to GOLD criteria. We estimated age– and gender–specific prevalence of COPD using a multilevel mixed–effect logistic regression. We also presented the time trends of COPD between 1990 and 2010 by age, gender and setting (urban vs rural).

**Findings:**

In 1990, the prevalence of COPD ranged from 0.49% (95% CI = 0.29–0.85) in <20 years group to 20.95% (95% CI = 14.04–27.04) in> = 80 years group, and the crude prevalence for China was 2.70% (95% CI = 1.86–3.51). In 2010, the prevalence in <20 years was 0.55% (95% CI = 0.37–1.04) and in> = 80 years was 22.89% (95% CI = 18.13–28.96), with the crude prevalence for China of 3.84% (95% CI = 3.30–4.77). The COPD prevalence in males was about two–fold higher than in females, and it increased with increasing age. Between 1990–2010, the total number of Chinese people living with COPD increased by 66.73%, from 30.90 million (95% CI = 21.28–40.02) in 1990 to 51.52 million (95% CI = 44.26–63.93) in 2010. This increase was most striking in middle age, and greater in females than in males from 30 years up to 64 years. Our estimates, which used an independent approach to acquiring data and development of analytical methods, and were based on a more complete data set, are remarkably similar to those produced recently by the GBD 2013 collaboration, differing by only about 5% in the estimated number of COPD cases in 1990 and by 1% in 2010.

**Conclusions:**

COPD is a highly prevalent disease in China and its importance is growing steadily. The number of people living with COPD has increased substantially between 1990 and 2010. COPD is more frequent in males and in rural areas. Optimised primary and secondary prevention and treatment is urgently needed to counter this growing trend. Improved epidemiological studies will be required to assist development of more effective strategies of prevention and treatment of COPD in China in the next decade and beyond.

Chronic obstructive pulmonary disease (COPD), characterized by progressive airflow limitation that is not fully reversible, is a major cause of chronic morbidity and mortality. It also carries a substantial financial burden on society [1–4]. It is estimated that COPD was the sixth most frequent cause of death worldwide in 1990, and will become the third by 2020 [5,6]. Within the same time frame, COPD is also projected to become the third largest cause of global morbidity [5,7]. Cigarette smoking is the major risk factor for COPD, and others include occupational exposures, air pollution, airway hyper–responsiveness and asthma, while genetic predisposition may also play some role [8–11]. The startling spread of this global health epidemic is occurring as a result of the continuous exposure to COPD risk factors and the general aging of the global human population [5,12,13]. In China, where the population is aging very rapidly as a result of improved living conditions, urbanization, high economic growth and family planning [14,15], COPD has become the leading cause of disability [16], resulting in a very large economic burden [17–19]. Furthermore, the Chinese population is also at high risk of developing respiratory diseases because of indoor and outdoor pollution [10,20,21]. All the aforementioned factors indicate that COPD may present a large challenge for the Chinese health care system in the foreseeable future.

Despite growing evidence of epidemiological and economic impact, the availability of accurate disease burden estimates for COPD in China is challenged by the fact that COPD is often underdiagnosed or misdiagnosed [1,11]. Up–to–date information about the prevalence of COPD in general population is essential to inform stakeholders and guide health services allocation [22]. However, the evidence on the prevalence of COPD in the Chinese population is quite inconsistent, probably due to variation in case definition and diagnostic methods, or study population characteristics [20,23]. The latest Global Burden of Disease (GBD) 2013 study revealed a prevalence of COPD of 7.3% (95% CI = 6.7%–7.9%) in the Chinese population aged 40 years and above in the year 2013 [23]. The GBD 2013 study only included studies of high quality, but the limited number of included studies on COPD prevalence in China could have also limited the authors' ability to address the burden across the whole country. In addition, COPD becomes progressively more common with age, and it could also develop relatively early in life, while previous studies in China mainly focused on middle–aged populations [24,25]. It is, therefore, worth exploring the burden of COPD across the entire lifespan. Another feature of COPD is that its prevalence is likely associated with the greatest socioeconomic inequality in comparison to other common diseases, as COPD is generally believed to disproportionally affect the poor [17,22,26]. In China, difference between the urban and rural setting provides a chance to explore the influence of socioeconomic factors and we are not aware of a systematic analysis of urban–rural differences at the national level.

Large Chinese bibliographic databases provide a considerable amount of information that should allow studying the epidemiology of disease in China [27,28]. In addition, spirometry method – which is the gold standard for the diagnosis of COPD [1,29] – has been widely adopted as an accurate estimate of the true burden of COPD in more recent epidemiological studies in China. This provides an opportunity to systematically assess the burden of COPD in China based on a standard case definition and diagnostic method. In this study, we conducted a comprehensive systematic review, in both Chinese and English databases, to analyze published population–based studies of COPD prevalence in China from 1990 onwards, based on spirometry as a diagnostic tool. We also presented the time trends of COPD between 1990 and 2010 by age, gender and setting (urban vs rural).

## METHODS

### Systematic review and data extraction

**Figure 1:**
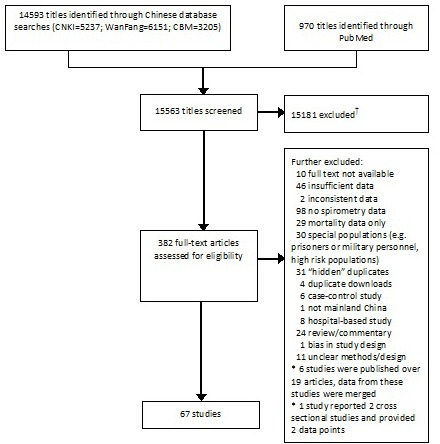
PRISMA diagram of study selection. † –The large majority was excluded because they were duplicate returns of the same reference under different search terms or in different databases; the others were excluded because they were irrelevant to the topic of our study, provided no numerical estimates, or studied Chinese populations outside of mainland China. * – These are duplicate publications of the same results in different journals.

We used CNKI, WanFang, CBM and PubMed academic databases to retrieve publications on COPD prevalence in China. Parallel searches of the databases were conducted by two independent trained reviewers (Chinese databases: XL and WC; English databse: KYC and ANP). The search was limited to studies published between 1990 and 2014 (**Figure 1**). The initial screening of the four databases returned 5237 results from CNKI, 6151 from the WanFang, 3205 from CBM, and 970 from PubMed, respectively. 382 studies were retained after excluding duplicates within and between databases, studies with no numerical values, studies conducted outside mainland China, review articles, viewpoints, conference abstracts. The process is documented in detail in **Figure 1**.

Further steps included obtaining full–text versions of the retained 382 articles and checking whether they met the minimum quality criteria to be used for further analyses and modelling. Studies were subsequently excluded if they were not prospective in design, not population based, did not report spirometry data on COPD prevalence, did not follow the Global Initiative for Chronic Obstructive Lung Disease (GOLD) standard of a fixed post–bronchodilator ratio of FEV1/FVC less than 0.7, did not have full text available, or contained insufficient or inconsistent data.

## Statistical analysis

In the data extraction procedure, individual studies were used to provide multiple data points which contributed to the overall data set. To take into account the sample size and the availability of different data points from the same study, a multilevel mixed–effect logistic regression was adopted with a restricted cubic spline [30,31]. This was then used to model the COPD prevalence as a function of age. Based on a total of 419 data points extracted from 67 studies with spirometry–based diagnosis of COPD, the gender– and age–specific prevalence of COPD in China was estimated. We further estimated the corresponding 95% confidence intervals (CIs) by applying the semiparametric bootstrapping method [32].

The number of gender–specific COPD cases in China in a population 20 years and older in a given year was calculated by multiplying the estimated gender–specific prevalence for each 5–year age group with the corresponding 5–year population in the same year, available from the United Nations Population Division (UNPD). This was performed for the years 1990, 2000 and 2010. Then, the gender–specific prevalence of COPD for the population aged <20 years was calculated by dividing the aggregated number of COPD cases observed in this age group (which was rather small) with the aggregated size of the corresponding population in this age group. This was done across the age groups 0–4, 5–9, 10–14 and 15–19 years old. The total number of people living with COPD was derived by adding together the numbers of males and females living with COPD, and the overall COPD prevalence was calculated by dividing the total number of COPD cases with the total size of the population in a given year. All the analyses were conducted in R v3.3.0 (R Development Core Team; http://www.R–project.org).

## RESULTS

The characteristics of the 67 retained cross–sectional studies are shown in Table S1 and Table S2 in **Online Supplementary Document(Online Supplementary Document)**: the studies were typically large, published mainly in the past decade and led by multi–disciplinary teams of specialists. Geographically, the studies covered all 31 provinces/municipalities/autonomous regions of mainland China.

**Figure 2:**
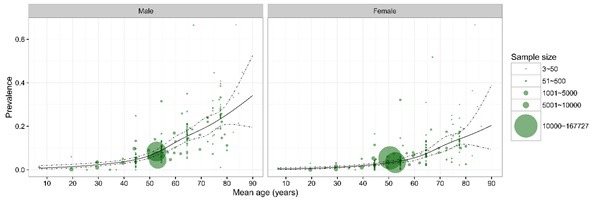
Gender and age–specific prevalence of chronic obstructive pulmonary disease (COPD) in China based on the information from the systematic review. The size of each bubble is proportional to the sample size, where at younger (<20 years) and older (>80 years) groups, the regression lines are based on fewer data points

**Figure 3:**
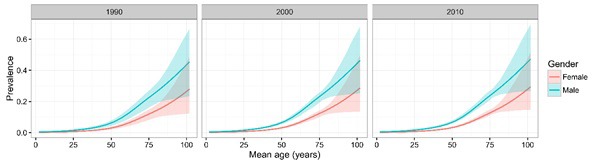
Chronic obstructive pulmonary disease (COPD) prevalence for males and female in the years 1990, 2000 and 2010, with 95% confidence intervals.

The 67 retained studies incorporated 759 461 people tested for COPD, 45 197 of whom were diagnosed with COPD using the spirometry method. Across most of the age spectrum in adulthood (20 years and more), there were large sample sizes providing substantial numbers of data points, except for the very old age (>80 years). A total of 47 studies provided the gender–specific prevalence information. **Figure 2** shows the gender–specific relationship between the COPD prevalence and age, based on all the informative data points. Generally, the prevalence of COPD increased steadily with age in both males and females. In males, it reached a prevalence of about 25% by the age of 80 years, and in females it was about twice less prevalent. Based on the information from the 47 studies, the estimates of COPD prevalence for males and females in the years 1990, 2000 and 2010 are shown in **Figure 3**. Across all three years, the prevalence of COPD was consistently greater in males than in females, and the difference was most pronounced in older adults.

**Figure 4:**
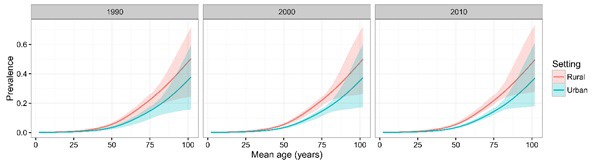
Chronic obstructive pulmonary disease (COPD) prevalence for urban and rural dwellers in the years 1990, 2000 and 2010, with 95% confidence intervals.

A total of 56 studies provided the setting–specific prevalence information. The comparison of COPD prevalence between urban and rural dwellers in the years 1990, 2000 and 2010 is presented in **Figure 4**. Similar to the comparison of gender–specific prevalence, there was a marked difference in COPD prevalence between urban and rural dwellers, where people in rural areas had higher COPD prevalence than those in urban areas.

The overall prevalence of COPD and the number of COPD cases were generated based on the UNPD demographic data, where no demographic information for urban and rural areas was provided, so the setting was not included in the final modelling process. The formula generated from the multilevel mixed–effect logistic regression is shown below:

ln(odds) = −13.388 + 0.004 × year +0.763 × gender +0.058 × age1 + 0.022 × age2 − 0.083 × age3 + 0.026 × age4+ui

Where:

odds = p/(1−p), p indicates the prevalence of COPD

year = calendar year

gender = 1 for males and gender = 0 for females

age1 –age4 are variables created in the process of fitting cubic splines (knots: 34.5, 49.5, 59.8, 71.7, 79.0)

ui = the study level random effect

**Figure 5:**
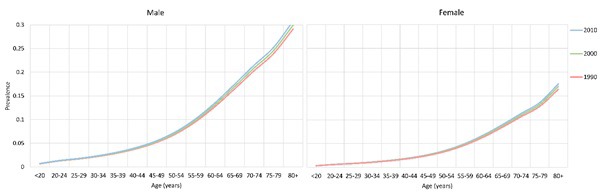
Estimated gender– and age–specific prevalence of chronic obstructive pulmonary disease (COPD) in China in the years 1990, 2000 and 2010.

After applying the estimates of age– and gender–specific COPD prevalence on UN Population Division's demographic data, the prevalence of COPD in populations aged <20 years and ≥80 years were calculated. From 1990 to 2010, the changes in the gender–specific COPD prevalence across all age groups are shown in **Figure 5** and **Table 1**. Over the 20 years considered in this analysis, the prevalence of COPD increased slightly in both males and females, which was also indicated in the model formula, where the annual rate of change in prevalence on a log odds scale was only 0.004. In 1990, the prevalence of COPD ranged from 0.49% (95% CI = 0.29–0.85) in younger people aged under 20 years to 20.95% (95% CI = 14.04–27.04) for those who were 80 years of age or older, and the overall prevalence was 2.70% (95% CI = 1.86–3.51). In 2010, the prevalence of COPD ranged from 0.55% (95% CI = 0.37–1.04) in people aged under 20 years to 22.89% (95% CI = 18.13–28.96) in people aged 80 years and above, with the overall prevalence in the Chinese population increasing to the level of 3.84% (95% CI = 3.30–4.77). The COPD prevalence between males and females differed consistently from 1990 to 2010, where the prevalence of COPD in males was around two–fold higher than in females, and this difference increased with increasing age. During 1990 to 2010, the overall prevalence of COPD increased by 42.22%. The most significant increases were in younger population who were under 20 years (12.24%) and older population aged 80 years and above (9.26%). The increasing rates were similar in males and females.

**Table 1:** Estimated gender– and age–specific prevalence of chronic obstructive pulmonary disease (COPD) in China in the years 1990 and 2010

	Prevalence of COPD in 1990 (%)			Prevalence of COPD in 2010 (%)			Rate of change (%, 1990–2010)		
**Age (years)**	**Male**	**Female**	**Overall**	**Male**	**Female**	**Overall**	**Male**	**Female**	**Overall**
<20	0.65 (0.38–1.14)	0.31 (0.18–0.54)	0.49 (0.29–0.85)	0.72 (0.49–1.36)	0.34 (0.23–0.65)	0.55 (0.37–1.04)	10.77	9.68	12.24
20–24	1.26 (0.83–1.86)	0.59 (0.40–0.87)	0.94 (0.62–1.38)	1.35 (1.06–2.11)	0.64 (0.50–1.02)	1.01 (0.79–1.59)	7.14	8.47	7.45
25–29	1.67 (1.15–2.33)	0.79 (0.55–1.09)	1.25 (0.86–1.73)	1.79 (1.46–2.59)	0.84 (0.69–1.25)	1.33 (1.09–1.93)	7.19	6.33	6.40
30–34	2.22 (1.56–2.93)	1.05 (0.74–1.39)	1.66 (1.17–2.19)	2.37 (2.00–3.20)	1.12 (0.95–1.53)	1.76 (1.49–2.38)	6.76	6.67	6.02
35–39	2.93 (2.09–3.79)	1.39 (0.99–1.80)	2.20 (1.57–2.84)	3.14 (2.73–4.00)	1.49 (1.31–1.93)	2.33 (2.04–2.98)	7.17	7.19	5.91
40–44	3.88 (2.71–5.01)	1.85 (1.29–2.37)	2.90 (2.03–3.75)	4.15 (3.60–5.15)	1.98 (1.75–2.48)	3.09 (2.70–3.85)	6.96	7.03	6.55
45–49	5.18 (3.60–6.53)	2.48 (1.71–3.17)	3.90 (2.71–4.94)	5.54 (4.83–6.71)	2.66 (2.35–3.26)	4.13 (3.62–5.03)	6.95	7.26	5.90
50–54	7.02 (4.92–8.75)	3.40 (2.33–4.29)	5.31 (3.70–6.65)	7.49 (6.66–8.84)	3.64 (3.26–4.30)	5.63 (5.02–6.65)	6.70	7.06	6.03
55–59	9.55 (6.71–11.92)	4.69 (3.25–5.97)	7.23 (5.06–9.08)	10.18 (8.88–11.97)	5.02 (4.41–5.98)	7.68 (6.72–9.07)	6.60	7.04	6.22
60–64	12.77 (9.00–15.64)	6.39 (4.36–7.98)	9.66 (6.74–11.91)	13.58 (11.98–15.74)	6.82 (5.99–8.06)	10.24 (9.02–11.95)	6.34	6.73	6.00
65–69	16.48 (11.69–19.91)	8.42 (5.73–10.45)	12.38 (8.66–15.09)	17.47 (15.40–19.97)	8.98 (7.83–10.52)	13.24 (11.64–15.27)	6.01	6.65	6.95
70–74	20.32 (14.30–24.44)	10.62 (7.16–13.33)	15.23 (10.56–18.62)	21.48 (18.59–24.84)	11.31 (9.65–13.47)	16.29 (14.03–19.04)	5.71	6.50	6.96
75–79	23.95 (17.27–28.67)	12.80 (8.91–15.90)	17.68 (12.57–21.49)	25.26 (22.67–28.42)	13.61 (12.02–15.65)	18.99 (16.94–21.55)	5.47	6.33	7.41
80+	29.10 (20.17–36.28)	16.38 (10.60–21.86)	20.95 (14.04–27.04)	30.80 (24.85–37.70)	17.51 (13.57–23.02)	22.89 (18.13–28.96)	5.84	6.90	9.26
Total	3.47 (2.40–4.49)	1.88 (1.28–2.47)	2.70 (1.86–3.51)	4.91 (4.22–6.06)	2.69 (2.31–3.37)	3.84 (3.30–4.77)	41.50	43.09	42.22

**Figure 6:**
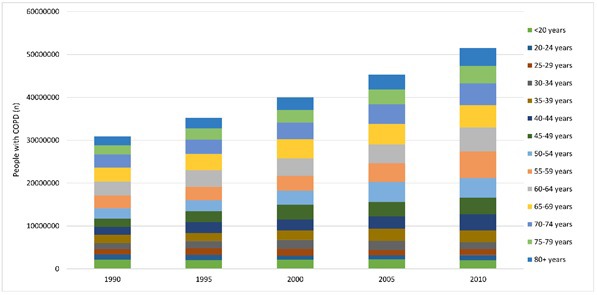
Estimated numbers of people living with chronic obstructive pulmonary disease (COPD) in China by year and age group.

The estimates of the number of people living with COPD in China are shown in **Table 2** and **Figure 6**. With the aging of the Chinese population during 1990–2010, the total number of Chinese people living with COPD increased by 66.73%, from 30.90 (95% CI = 21.28–40.02) million in 1990 to 51.52 (95% CI = 44.26–63.93) million in 2010. Throughout this period, the number of COPD cases decreased by 7.41% in younger people aged under 20 years. The most significant increases were in those who were middle–aged (40–49 years and 55–59 years) and at older ages (80 years and above), with rates of change in the absolute number of cases in those age–groups above 100%. This increase was greater in females than in males from 30 years up to 64 years. In 2010, around two–thirds (66.34%) of the people living with COPD were males in China.

**Table 2:** Estimated numbers of people living with COPD in China in the years 1990 and 2010, and the rate of change from 1990 to 2010, by gender and age group

	People living with COPD in 1990 (million)			People living with COPD in 2010 (million)			Rate of change (%, 1990–2010)		
**Age (years)**	**Male**	**Female**	**Overall**	**Male**	**Female**	**Overall**	**Male**	**Female**	**Overall**
<20	1.50 (0.88–2.61)	0.66 (0.39–1.15)	2.16 (1.28–3.77)	1.44 (0.98–2.72)	0.57 (0.39–1.09)	2.00 (1.37–3.80)	–4.00	–13.64	–7.41
20–24	0.83 (0.55–1.22)	0.36 (0.24–0.53)	1.19 (0.79–1.75)	0.85 (0.66–1.32)	0.36 (0.28–0.58)	1.21 (0.95–1.91)	2.41	0.00	1.68
25–29	0.86 (0.59–1.19)	0.38 (0.26–0.52)	1.23 (0.85–1.71)	0.93 (0.76–1.34)	0.41 (0.34–0.61)	1.34 (1.10–1.95)	8.14	7.89	8.94
30–34	1.00 (0.70–1.32)	0.43 (0.30–0.57)	1.42 (1.00–1.88)	1.12 (0.94–1.51)	0.50 (0.43–0.68)	1.62 (1.37–2.19)	12.00	16.28	14.08
35–39	1.36 (0.97–1.75)	0.58 (0.41–0.75)	1.94 (1.38–2.50)	1.91 (1.66–2.43)	0.87 (0.77–1.13)	2.78 (2.43–3.56)	40.44	50.00	43.30
40–44	1.28 (0.90–1.66)	0.56 (0.39–0.72)	1.85 (1.29–2.38)	2.60 (2.26–3.22)	1.17 (1.04–1.47)	3.77 (3.29–4.70)	103.13	108.93	103.78
45–49	1.34 (0.93–1.69)	0.58 (0.40–0.74)	1.92 (1.33–2.43)	2.68 (2.33–3.24)	1.23 (1.08–1.50)	3.90 (3.42–4.74)	100.00	112.07	103.13
50–54	1.69 (1.19–2.11)	0.73 (0.50–0.92)	2.42 (1.69–3.03)	3.13 (2.79–3.70)	1.42 (1.27–1.67)	4.55 (4.06–5.37)	85.21	94.52	88.02
55–59	2.08 (1.46–2.60)	0.93 (0.65–1.19)	3.01 (2.11–3.78)	4.24 (3.70–4.98)	1.96 (1.72–2.33)	6.19 (5.42–7.31)	103.85	110.75	105.65
60–64	2.21 (1.56–2.71)	1.05 (0.72–1.32)	3.27 (2.28–4.03)	3.80 (3.35–4.40)	1.86 (1.64–2.20)	5.66 (4.99–6.61)	71.95	77.14	73.09
65–69	2.10 (1.49–2.53)	1.11 (0.76–1.38)	3.21 (2.24–3.91)	3.42 (3.02–3.91)	1.74 (1.52–2.04)	5.16 (4.54–5.95)	62.86	56.76	60.75
70–74	1.92 (1.35–2.31)	1.11 (0.75–1.39)	3.03 (2.10–3.71)	3.25 (2.81–3.75)	1.78 (1.52–2.12)	5.03 (4.33–5.87)	69.27	60.36	66.01
75–79	1.30 (0.94–1.55)	0.89 (0.62–1.11)	2.19 (1.56–2.66)	2.54 (2.28–2.86)	1.59 (1.41–1.83)	4.14 (3.69–4.70)	95.38	78.65	89.04
80+	1.03 (0.71–1.28)	1.03 (0.67–1.38)	2.06 (1.38–2.66)	2.27 (1.83–2.78)	1.90 (1.47–2.50)	4.17 (3.30–5.27)	120.39	84.47	102.43
Total	20.50 (14.22–26.53)	10.40 (7.06–13.67)	30.90 (21.28–40.02)	34.18 (29.37–42.16)	17.36 (14.88–21.75)	51.52 (44.26–63.93)	66.73	66.92	66.73

## DISCUSSION

In this study, by systematically reviewing all published evidence of COPD prevalence databased on spirometry, and applying strict inclusion and exclusion criteria, we presented comprehensive and data–driven estimates of the COPD prevalence in China across the entire age range, and the differences between males and females, and urban and rural dwellers in China. As suggested by the Global Initiative for Chronic Obstructive Lung Disease (GOLD), a fixed post–bronchodilator ratio of FEV1/FVC less than 0.7 was used as the primary indicator of COPD diagnosis in our study [1]. In the present study, we estimated a national GOLD–defined COPD prevalence of 2.70% in 1990 and 3.84% in 2010, corresponding to 30.90 million and 51.52 million people living with COPD in 1990 and 2010 respectively. Our estimate for the year 2010 was similar to the estimate presented in the GBD 2013 study, which reported 32.44 million of cases in 1990 and 54.79 million of cases in 2013 [23]. Given that the estimates presented in GBD 2013 study were based on a limited number of studies that were mostly published after 2000, any differences in compared COPD number of cases for the year 1990 between these two studies – amounting to 1.54 million (or about 5% difference) – may be explained through this lack of data in the GBD 2013 study. Our estimate in 2010 is relevant to a point in time 3 years earlier than the GBD 2013 estimate, but based on our time trends we would expect our estimate for 2013 to be nearly identical to the GBD estimate, ie, just over 54 million cases (with difference of about 1%). In comparison with the results of the GBD 2013 study, where a slight decrease of a standardized COPD prevalence was reported between 1990 and 2013 (from 3.7% to 3.6% overall prevalence in the population), our study observed an overall increase trend of COPD prevalence during the two decades between 1990 to 2010 – from 2.70% to 3.84%. Our estimates were much in line with the increasing trend of COPD prevalence globally and in the Western Pacific region [4,11]. Further analysis from an epidemiological perspective will remain to be required to explore the temporal distribution of COPD prevalence in China. The detailed and systematic estimates of COPD prevalence and the number of cases in this study constitute the best currently available basis for policymaking, planning, and allocation of health and welfare resources related to the burden of COPD in China. The strength of this study arises from several measures that we took to derive the estimates. First, we did a comprehensive systematic review and thus included all available studies. Second, of all the measurement methods, we chose spirometry method as the diagnosis criteria, which served to avoid potential bias arising from the variation of measurement tools. Third, the estimate of prevalence was based on study points that came from many different locations China, which should ensure representativeness for the whole nation.

In view of the relation between COPD prevalence and age, our study confirms that COPD is a progressive and degenerative disease [11,33]. By estimating the prevalence of COPD in the Chinese population over the full age–span, we enabled a public health approach to prevention and treatment of COPD in younger population. It was important to note that COPD can also exist in early life, as suggested by previous studies [24,25]. Younger populations should therefore not be excluded from COPD screening programs. In our study, although the number of COPD people in <20 years decreased slightly during 1990 to 2010, the prevalence rate kept increase. Early diagnosis is very important, and disease–modifying therapies that can delay onset could have considerable potential for reducing age–specific prevalence [34].

As expected, the prevalence in males in China was much higher than in females. The excess was most likely to be explained by historic patterns of smoking and occupational exposures in Chinese men [4,20,35]. From a physiological perspective, it is also widely hypothesized that women are more vulnerable to the lung–damaging effects of cigarette smoking and biomass fuels, and more prone to develop COPD than men when they are at the same level of hazard exposure [35–37]. Our study showed a slightly higher increase of COPD prevalence and of the number of affected individuals in females than in males during 1990 and 2010. Based on this trend, it should be expected that prevalence of COPD in Chinese women is set to rise in the coming years, partly because of a markedly increasing rate of cigarette smoking in Chinese women [38,39].

The prevalence of COPD was consistently higher in rural areas than in urban areas in our study. This was in contrast with the global estimates, which show that COPD is more frequent in urban areas [4,40]. The possible explanation is the variation in classification of rural and urban settings across the world. In China, rural areas are generally those with less (or lower–quality) health resources and worse health outcomes in comparison to urban areas [41–44]. This urban–rural disparity of COPD prevalence may be associated with higher smoking rates, but also with prevalent indoor air pollution resulting from the burning of wood and other biomass fuels in rural populations [10,26,45]. In addition, lower socioeconomic status, lower health resources quality, and worse quality of cigarettes may also have contributed [20,21,26].

A striking increase of 66.73% in the absolute number of estimated COPD cases between 1990 and 2010 in China was indicated by our analysis. This increase was most prominent in middle–aged and older populations. The primary drivers of this increase were longer life expectancy in China, which led to an increase of the number of individuals in older age groups, and continuous exposure to risk factors, which led to an increase in prevalence among the middle–aged. The estimated number of 51.52 million people living with COPD in China in 2010 represents a very large burden for both China and the world, where an estimated number of 328 million people have COPD [46,47]. With the current trends of ageing in China [14,15], the numbers of individuals affected with COPD will continue to increase, particularly in older population. Elderly with medical care needs will constitute a burden on the health care system, and support for family caregivers may also be needed [14,44]. In young and middle–aged populations, the limitations in their ability to work and their overall mobility may not only lead to a reduced quality of life, but also bring an economic challenge to both individuals and the society [18–20]. Even among the affected individuals who only have mild symptoms, the effects of COPD may result in impaired quality of life. These effects might be profound in many poor settings, where continuous exposure to risk factors could accelerate the progress of the disease process [48,49].

Our study also had several potential shortcomings. Significant heterogeneity was seen between all of our included studies. This is not surprising, given that China is a diverse country with highly varied culture, levels of development and demographic characteristics. Although only studies that adopted the spirometry method and the GOLD criterion were included, in order to reduce the methodological variability, heterogeneity may still exist because of the differences among investigators in operating protocols, levels of training, adherence to guidelines and variations in implementation of the guidelines. Another possible cause for concern is that the results of meta–regression only took into account a limited set of covariates (ie, gender, age and urbanicity) because these were the only covariates that were broadly available. The scarcity of studies adopting unified definition of other covariates limited our ability to explore more possible effects. Our estimates, therefore, do not take into account the role of special sub–groups exposed to risk factors, such as smokers, household members who are exposed to biomass fuels used for home heating and cooking, workers with occupational exposures to dust, and people with tuberculosis. This is a shortcoming that needs to be addressed in future epidemiological studies of COPD in China. In addition, the estimates of prevalence in younger and older age groups were clearly less certain due to scarce data and require further refinement. Finally, although GOLD criteria are widely accepted as a diagnostic tool for COPD, they are prone to false–negative results among younger and false–positive among older adults, which should be taken into account [1,26]. We were also unable to account for the severity of COPD, particularly to characterise the burden of the early–stage COPD.

A growing amount of evidence suggests that ambient air pollution is a risk factor for COPD [10,50]. This hypothesis may be of particular interest in future COPD epidemiology studies in China, where a dramatic increase in emissions of ambient air pollutants has become a large societal concern [51]. Concern is often focused on observed air pollution levels in very large cities, but our analysis didn't find support for COPD prevalence being a larger problem in urban as opposed to rural areas.

Our work has implications in both the academic and public health areas. Epidemiological studies that are typically available in China on COPD could be designed better, with standardized diagnostic criteria and much better presentation of results, taking into account different covariates. Present gaps would ideally be filled by a national disease surveillance system, but this would need considerable and sustainable funding and training. Perhaps a more realistic approach would include high–quality epidemiology studies in large samples, such as Kadoorie study – which provided a large portion of all the data available for our study in terms of sample size [52,53]. Adopting standardized measures of COPD, with accurate assessments of the stage and severity of COPD, effectiveness of treatment and clear definition of study population sub–groups in relation to their risk exposure would all help. Currently, most people with COPD can only receive a diagnosis in the late stage of disease when symptoms are rather obvious [18]. Although COPD is not a fully reversible disease, the benefits of earlier diagnosis and treatment have been well established [11,18]. Efforts should be taken to improve the quality and availability of health care resources to address COPD both from the point of prevention, diagnosis and treatment, especially in rural areas where the rates of COPD prevalence are higher. In the meanwhile, the importance of primary prevention should also be addressed, especially in primary care settings. Health education should focus on targeted populations based on the existing evidence [21]. Advocated measures include implementation of tobacco–free policies, adequate treatment of asthma and comprehensive strategies on reducing indoor pollution. [11,18,21].

## CONCLUSIONS

In conclusion, results from this study have shown that COPD is a highly prevalent disease in China and its importance is growing steadily. The number of people living with COPD has increased substantially and nearly doubled between 1990 and 2010. COPD is more frequent in males and in rural areas. Optimal intervention delivery – both in primary and secondary prevention and treatment – is urgently needed to counter this growing trend. Improved epidemiological studies will be required to assist development of more effective strategies of prevention and treatment of COPD in China in the next decade and beyond.
