# Supplementary material for: Prevalence of chronic obstructive pulmonary disease (COPD) in China in 1990 and 2010
Source: J Glob Health. 2017 Dec 20;7(2):020704. doi: 10.7189/jogh.07.020704 (PMC5785871; doi:10.7189/jogh.07.020704)
Supplement: Online Supplementary Document [file jogh-07-020704-s001.pdf]

Prevalence of Chronic Obstructive Pulmonary Disease (COPD) in China in 1990 and 2010

Table S1. Full list of the included studies on COPD prevalence in China (n=67)

Study ID	Province	Reference
S1	Beijing	Yao WZ, Zhu H, Shen N, Han X, Liang YJ, Zhang LQ, Sun YC, Hao ZT, Zhao MW (姚婉贞, 朱红, 沈宁, 韩翔, 梁岩静, 张立强, 孙永昌, 郝振婷, 赵鸣武). Epidemiological data of chronic obstructive pulmonary disease in Yanging County in Beijing (北京市延庆县慢性阻塞性肺疾病流行病学). Journal of Peking University (Health Sciences). 2005; 37(2): 121-125.
S2	Beijing	Chen QH, Kou ZF, Pan N, Wang LX, Zheng Y (陈乾华, 寇志芳, 王立新, 郑野). Survey on incidence of chronic obstructive disease in the northern area of Beijing (北京朝阳区慢性阻塞性肺疾病的流行病学调查). China Tropical Medicine (中国热带医学杂志). 2010; 10(9): 1086 and 1094.
S3	Beijing	Zhou WL, Du XP (周文利, 杜雪平). Morbidity rate of COPD among high risk population in the community (社区慢性阻塞性肺疾病高危人群的患病状况调查). Chinese General Practice (中国全科医学). 2011; 14(19): 2197-2204.
S4	Beijing	Cheng Y, Zhao YN, Liu JQ, Kang H, Xia GG, Hu P, Zhang C, Wang GF (程渊, 赵燕妮, 李楠, 刘俊青, 康卉, 夏国光, 胡萍, 宿利, 张成, 王广发). The investigation of COPD diagnostic situation in an urban community of Beijing (北京市区慢性阻塞性肺疾病诊断现状调查). Chinese Journal of Medicine (中国医刊). 2011; 46(4): 46-49.
S5	Beijing	Li JZ, Xu XS, Xie BY, Hao FX, Zhang XG (李景周, 徐希胜, 谢宝元, 郝凤霞, 张学功). An investigation into pulmonary heart disease among 50,000 residents in Fangshan district, Beijing *(北京房山地区 5 万人群肺心病调查分析). Chinese Journal of Prevention and Control of Chronic Non-Communicable Diseases (中国慢性病预防与控制). 1996;4(4):181-182.
S6	Chongqing	Li Q, Liao XQ, Zhang Q, Wang Y, Wu XL, Xu Z, Jiang H, Luo QH, Kong DP, Zhao ZQ, WS,Wang JP, Qian GS, Wang CZ (李琦, 廖秀清, 张巧, 王彦, 吴学玲, 徐智, 江汉, 罗庆红, 孔德平, 赵志强, 王金平, 钱桂生, 王长征). Epidemiologlcal sampling survey on chronic obstructive pulmonary disease in urban area of Chongqing (重庆市部分城区慢性阻塞性肺疾病流行病学的抽样调查). Chinese Journal of Respiratory and Critical Care Medicine (中国呼吸与危重监护杂志). 2009; 8(1): 12-15.
S7	Chongqing	Weng HA, Lai FH, He ZJ, Gou Q, Xu XX, Tan GG, Yang MQ, Yin XY, Liu DP, Li XY, Wang Z, Hu Y, Chen QR, Cheng X (翁航爱,赖富华,何治军,勾琼,徐新献,谭光根,杨明清,尹晓燕,刘大鹏,李夏渝,王忠,胡忆,陈清荣,程秀). Epidemiological

		Survey On COPD in part of the Chongqing Communitis* (重庆市部分城市社区 COPD 流行病学调查). The fifth national conference on chronic obstructive pulmonary disease (第五届全国慢性阻塞性肺疾病学术会议). 106.
S8	Guangdong	Wang DL, Liu SM, Zhou YM, Lu JC, Zheng JP, Zhong NX, Ran PX (王大礼,刘升明,周玉民,吕嘉春,郑劲平,钟南山). Epidemic analysis of COPD in Liwan district of Guangzhou city (广州荔湾区 COPD 流行病学调查分析). Academic Journal of Guanghou Medical College(广州医学院学报). 2004;32(2):42-44.
S9	Guangdong	Liu SM, Wang XP, Wang DL, Zhou YM, Lv JC, Zheng JP, Zhong NS, Ran PX (刘升明, 王小平, 王大礼, 周玉民, 吕嘉春, 郑劲平, 钟南山, 冉丕鑫). An analysis of the epidemiology of COPD in Guangdong Province* (广东部分地区慢性阻塞性肺疾病发病状况调查). National Medical Journal of China (中华医学杂志). 2005; 85(11): 747-752.
S10	Guangdong	Weng JL, Zheng YS, Wang WL, Guan LY, Ma QF (翁俊良, 郑义珊, 王文莉, 官玲燕, 马琼凤). An investigation into the Prevalence of COPD in Shantou area* (汕头地区慢性阻塞性肺疾病患病情况的调查). Hainan Medical Journal (海南医学). 2006; 17(12): 122-123.
S11	Guangdong	Su WQ, Zhou YM, Chen H, Chen XT, Liu SM, Lin HB, MO JD, Liu YH, Huang YH, Li DY, Zheng JP, Lv JC, Zhong NS, Ran PX (苏伟强, 周玉民, 陈虹, 陈秀桃, 刘升明, 林辉斌, 莫俊德, 刘宇晖, 黄耀光, 李帝吟, 郑劲平, 吕嘉春, 钟南山, 冉丕鑫). The difference between the prevalence of COPD between rural and urban areas among residents aged above 40 in Zhanjiang area* (湛江地区 40 岁以上人群 COPD 患病率的城乡差异). The Journal of Practical Medicine (实用医学杂志). 2007; 23(3): 310-313.
S12	Guangdong	Cai XZ, Lai SH, He CS (蔡孝桢, 赖书华, 何纯生). An analysis on the epidemiology and prevention and treatment of COPD in ruran Dongguan* (东莞农村地区 C OPD 流行病学调查及防治分析). International Medicine and Health Guidance News (国际医药卫生导报). 2012; 18(2):151-153.
S13	Guangdong	Li ZW, Chen J, Li XM (黎梓雯, 陈健, 李雪梅). Surveys and analysis of conditions of community residents with chronic pulmonary Disease (社区居民慢性阻塞性肺病状况调查及分析). Lingnan Journal of Emergency Medicine (岭南急诊医学杂志). 2009; 14(4): 257-258.
S14	Guangdong	Zhou YM (周玉民). An study on methoion oof epidemiology and prevention of COPD (慢性阻塞性肺疾病的流行病学调查方法与社区防治研究). Doctor's Thesis of Guangzhou Medical University. 2007.
S15	Guizhou	Ma LY, Dong ZQ, Wu KJ, Pan J (马利英,董泽琴,吴可嘉,潘军). Solid fuel use and health: a survey of rural women in Guizhou in winter (贵州农村冬季固体燃料使用与妇女健康调查). Environment Protection and Technology (环保科技). 2013;19(1):4-9.

S16	Hebei	Yu CL, Zhao CM, Li XX, Gao CJ, Yun L, Yao ZY, Xu YJ, Wang HY (喻昌利, 赵春梅, 李晓旭, 高长俊, 运玲, 么作义, 徐应军, 王红阳). Survey on prevalence of chronic obstructive pulmonary disease in rural areas of Tangshan (唐山农村地区慢性阻塞性肺疾病患病率调查). Clinical Focus (临床荟萃). 2009; 24(21): 1857-1860.
S17	Heilongjiang	Li HY (李海瑛). An analysis and preventive strategies of COPD* (慢性阻塞性肺病的患病因素及防治措施). Chinese Journal of Modern Drug Application (中国现代药物应用). 2009; 3(12): 74.
S18	Heilongjiang	Tang WL, Yu BQ, Zhang XF, Yan CH, Shao YX (唐文丽, 于百全, 张晓飞, 焉春华, 邵玉霞). Epidemiological survey of chronic obstructive pulmonary disease in Harbin rural areas (哈尔滨农村地区慢性阻塞性肺疾病流行现状调查). Journal of Clinical Internal Medicine (临床内科杂志). 2012; 29(12): 817-819.
S19	Heilongjiang	Yang CC, Wu XM, Wang XY, Kang XW, Huang K, Li ZG, Yang SN, Lin L (阳成成, 吴晓梅, 王欣燕, 康小文, 黄坤, 李兆国, 杨晟楠, 林琳). Epidemiological survey on chronic obstructive pulmonary disease in Harbin communities (哈尔滨市社区慢性阻塞性肺疾病流行病学调查). International Journal of Respiration (国际呼吸杂志). 2013;33(7):526-528.
S20	Hubei	Jiang RG, Luo DS, Huang CP, Li WM (蒋汝刚, 罗德生, 黄翠萍, 李伟明). Study on the Prevalence rate and risk factors of chronic obstructive pulmonary disease in rural community population in Hubei Province (湖北省部分农村地区人群慢性阻塞性肺疾病患病率及危险因素研究). Chinese Journal of Epidemiology (中华流行病学杂志). 2007; 28(10): 976-979.
S21	Hubei	MA J. Prevalence of COPD abd ECB Detection in Estimating of Airway Inflammation (thesis). Wuhan: Huazhoug University of Science and Technology, 2009.
S22	Hunan	Hong XQ (洪秀琴). Epidemic situation and risk factors analysis of COPD in partial areas of Hunan Province (湖南省部分地区慢性阻塞性肺疾病流行现状和危险因素分析). Master Thesis of Central South University. 2009.
S23	Hunan	Fu X, Hu H, Hu MF (付翔,胡红,胡敏凡). Epidemiological sampling survey on chronic obstructive pulmonary disease in urban area of Chenzhou city (郴州市部分城区慢性阻塞性肺疾病流行病学的抽样调查). Journal of Clinical Pulmonary Medicine (临床肺科杂志). 2011; 16(2): 184-185.
S24	Jilin	Jin SG, Zhang JP, Shi L, Wang HT, Li XJ (靳曙光, 张健萍, 石磊, 王洪涛, 李小军). Risk factors about COPD in some rural areas in Jilin City (吉林市部分农村慢性阻塞性肺疾病调查及危险因素分析). Chinese Rural Health Service Administation (中国农村卫生事业管理). 2009; 29 (7): 547-549.
S25	Liaoning	Li M (李猛). Epedemiologic investigation of risk factors of COPD in Peripheral villages of Shenyang (沈阳市周边农村慢性阻塞性肺疾病危险因素的流行病学研究). Postgraduate Thesis of China Medical University. 2005.

S26	Liaoning	Chen P, Zhao HT, Liu L, Sun L, Chi XL, Ma Z, Wang SY (陈萍,赵海涛, 刘蕾, 孙丽, 迟秀丽, 马壮, 王淑云). The investigation on the COPD morbility in communities of Shenyang (沈阳市部分社区慢性阻塞性肺疾病发病情况调查分析). Chinese Journal of Practical Internal Medicine (中国实用内科杂志). 2010; 30(3):227-229.
S27	Liaoning	Liu S, Wen DL, Li LY, Li ZH, Wang XG (刘朔, 闻德亮, 李丽云, 李振华, 王笑歌). An epidemiological study of chronic obstructive pulmonary disease in greenhouse farmers in Liaoning Province from 2006 to 2009 (2006-2009 年辽宁省大棚作业农民慢性阻塞性肺疾病的患病率调查). Chinese Journal of Tuberculosis and Respiratory Diseases (中华结核和呼吸杂志). 2011;34(10):753-756.
S28	Liaoning	Sun LL (孙丽丽). The relationship between smoking and prevalence of COPD of people aged 35 or older in some communities of Shenyang City (沈阳市部分社区 35 岁以上人群吸烟情况与 COPD 患病关系的调查). Master's Thesis of China Medical University. 2011.
S29	Qinghai	Zhou MR (周敏茹). An analysis on the prelance of chronic diseases in Xining area * (西宁地区居民慢性病患病现况分析). Journal of Qinghai Medical College (青海医学院学报). 2003; 24(4): 218-221.
S30	Qinghai	Gao F, Du FM, La Z, Yang CL (高芬, 杜发茂, 拉周, 杨彩铃). Epideioloical data of chronic cotpulmonale in patients with chronic obstructive pulmonarydisease in Qinghai (青海高原慢性阻塞性肺疾病所致慢性肺源性心脏病流行病学). International Journal of Respration (国际呼吸杂志). 2011; 31(13): 989-992.
S31	Shandong	Xiao YL, Li HY, Lin Y, Gao XC, Kong DZ, Liu RJ, Sui YN (肖耀来, 李合友, 林勇, 高宪成, 孔德众, 刘瑞娟, 隋云南). Study on prevalence and correlation factors of chronic obstructive plllmonary disease (慢性阻塞性肺疾病患病危险因素分析). Chinese Journal of Behaviour Medical Science (中国行为医学科学). 2005; 14(9): 786-787.
S32	Shandong	Li TX, Sun BX, Wang ZQ, Leng DN, Gao YX, Zhang KJ, Fang DH, Bai LY, Qin XY (李同新, 孙宾先, 王志庆, 冷大南, 高远翔, 张克江, 方东晖, 白霖宇, 秦学勇). Epidemiological Study on the Correlative Risk Factors for Chronic Obstructive Pulmonary Disease in Zhaoyuan (招远市农村慢性阻塞性肺疾病危险因素分析). Chinese Journal of Prevention and Control of Chronic Non-communicable Diseases (中国慢性病预防与控制). 2006; 14(5): 330-332.
S33	Shandong	Zhang MY (张明泳). An investigation into the risk factors of COPD in rural areas in Qingdao* (青岛市周边农村慢性阻塞性肺疾病危险因素的流行病学研究). Master Thesis of Qingdao University. 2006.
S34	Shandong	Zhang MY, Diao YQ (张明泳, 刁玉荃). An investigation of risk factors of chronic obstructive pulmonary disease (慢性阻塞性肺疾病危险因素的调查). Medical Journal of Qilu (齐鲁医学杂志). 2007; 22(5): 441-447.

S35	Shandong	Liu YH, Kong WS (刘聿慧, 孔维顺). A prevalence survey on chronic obstructive pulmonary disease among people aged over 60 years in rural Zaozhuang of Shandong province (山东省枣庄市农村地区 60 岁以上人群慢性阻塞性肺疾病患者病率调查). Chinese Journal of Natural Medicine (中国自然医学杂志). 2010; 12(6): 430 - 432.
S36	Shandong	Wang C (王川). An Analysis of COPD Epidemiological Status For Urban Residents in Jinan (济南市城市居民慢性阻塞性肺病流行病学调查分析). Shandong University Master's Thesis. 2010.
S37	Shandong	Li ZJ, Zhang SZ, Kong XT, Li ZH, Li JL, Qian AH, Xu JL (李兆金, 张守贞, 孔祥太, 李子洪, 李家岭, 钱爱华, 徐家莲). A prevalence survey on chronic obstructive pulmonary disease among people aged over 40 years in Zaozhuang rural area of Shandong province (山东省枣庄市农村地区 40 岁以上人群慢性阻塞性肺疾病患病率调查). Chinese Journal of Practical Medicine (中国实用医刊). 2011;38(1):19-24.
S38	Shandong	Wang LP (王立鹏). A analysis of the risk factors of Chronic Obstructive Pulmonary Disease among 422 rural residents* (422 例农村居民慢性阻塞性肺病的危险因素调查分析). Ghina Health Care & Nutrition (中国保健营养). 2012;7:2241-2242.
S39	Shandong	Zou DQ, Zhang LY, Sun WH, Wang Y, Gao H, Zhao JW, Zhang XS (邹德奇, 张鲁阳, 孙卫华, 汪瑜, 高海, 赵建伟, 张学顺). Current status in the prevention and management of chronic obstructive pulmonary disease in residents of Haiyang Shandong province (山东省海阳市居民慢性阻塞性肺疾病患病). Chinese Journal of Nautical Medicine and Hyperbaric Medicine (中华航海医学与高气压医学杂志). 2013;20(2): 107-110.
S40	Shanghai	Shen YE, Ye TT,ZHOU QM,Dai GQ,Fu H,Zhu JL,Huang JX,Zhou SH,Huang DY,Ding JQ,Lu PL（沈贻谔,叶葶葶,周群敏,戴国强,傅华,朱勒良,黄靖雄,邹四海,黄德渔,丁健琴,陆培廉). The current status of COPD in rural Shanghai (上海农村慢性阻塞性肺病研究综合报告). ACTA Academic Medicine Shanghai (上海医科大学学报). 1993; 20(suppl):132-136.
S41	Shanghai	(a)Shen YE, Ye TT, Fu H,Huang JX, Li SY,Gao YZ（沈贻谔,叶葶葶,傅华,黄靖雄,李守义,高玉珍). The potential risk factors for COPD among elderly residents in urban area of Shanghai (上海市区中老年人慢性阻塞性肺病的危险因素分析). Chinese Journal of Public Health (中国公共卫生学报). 1993; 12(6):360-362.
S42	Shanghai	(b)Shen YE, Ye TT, Fu H,Huang JX, Li SY,Gao YZ（沈贻谔,叶葶葶,傅华,黄靖雄,李守义,高玉珍). The potential risk factors for COPD among elderly residents in urban area of Shanghai (上海市区中老年人慢性阻塞性肺病的危险因素分析). Chinese Journal of Public Health (中国公共卫生学报). 1993; 12(6):360-362.
S43	Shanghai	Ma R, Cheng QJ, Yao D, Tao M, Ni JH, Zhou M, Wan HY, Huang SG (马睿, 程齐俭, 姚迪, 陶敏, 倪瑾华, 周敏, 万欢英, 黄绍光). Epidemiological survey of chronic obstructive pulmornary disease in the elderly in Shanghai (上海地区老年人慢性阻

		塞性肺部疾病的流行病学研究). Academic Journal of Shanghai Second Medical University (上海第二医科大学学报). 2005; 25(5): 521-524.
S44	Shanghai	Jiang SF, Zhu SZ, Zhou Y, Ruan MJ, Lu J, Sun Y, Zeng H (江孙芳, 祝墡珠, 周云, 阮美娟, 陆嘉, 孙宇, 曾恒). The Preliminary Investigation of Chronic Obstructive Lung Disease in community (社区慢性阻塞性肺疾病患病情况的初步调查). Clinical Medical Journal of China (中国临床医学). 2005; 12(5): 780-781.
S45	Shanghai	Zhang BY, Gu XZ, Xu RY, Yan JH, Gong Y, Wang XP, Chen QM, Lin LP, Han YR, Yao YL (张柏膺, 顾学章, 许仁勇, 严峻海, 龚燕, 王学鹏, 陈清妹, 林乐平, 韩影如, 姚仪立, 肖慧英). Epidemiological study of community chronic obstructive pulmonary disease and evaluation of its systemic interference (社区 COPD 的流行病学调查及干预性防治的疗效观察). Journal of Clinical Pulmonary Medicine (临床肺科杂志). 2007; 12(8): 841-843.
S46	Shanghai	Zhao MZ, Zhou X, Xu WG (赵勉之, 周新, 徐卫国). A study of prevention and treatment of chronic obstructive pulmonary disease in community (慢性阻塞性肺病社区综合防治状况研究). Journal of Clinical Medicine in Practice (实用临床医药杂志). 2009; 13(3): 7-16.
S47	Shanghai	Gong Y, Shi GC, Wan HY, Li M, Li QY, Cheng QJ, Yang K, Tang W, Xiang Y, Liu JL, Dai RR, Ni L (龚益,时国朝,万欢英,李敏,李庆云,程齐俭,杨昆,汤葳,项轶,刘嘉琳,戴然然,倪磊). Changes in prevalences of COPD with in 5 years in people aged no less than 60 years in Shanghai urban area (上海城区 60 岁以上人群 5 年内 COPD 患病率变化及原因分析). Journal of Shanghai Jiaotong University(Medical Science) (上海交通大学学报（医学版)). 2011; 31(2): 216-220.
S48	Shanghai	Cheng Y, Cheng LY, Wang YP, Lu Q, Pan DJ, Cheng ZQ (陈晔, 陈利云, 王燕萍, 陆晴, 潘桂俊, 陈志强). An analysis on the risk factors and interventions of COPD in community hospitals in Shanghai* (上海社区医院慢性阻塞性肺疾病危险因素的调查分析及干预策略). Shanxi Medical Journal (山西医药杂志). 2012;41(7):664-666.
S49	Shanghai	Xie J, Shi JD, Deng XQ, Long W, Li SQ, Gao YY (谢娟, 施劲东, 邓星奇, 龙威, 李善群, 高育瑶). Investigation of risk factors for chronic obstructive pulmonary disease in Jiangchuan street, Minhang district of Shanghai (上海市闵行区江川地区慢性阻塞性肺疾病发病情况和危险因素的调查分析). International Journal of Respiration (国际呼吸杂志). 2010;30(13):769-772.
S50	Shanghai	Han X (韩雪). Epidemiologic analysis of chronic obstructive pulmonary disease in community residents in Yangpu District of Shanghai (上海市杨浦区社区居民慢性阻塞性肺疾病流行病学分析). Health Education and Health Promotion (健康教育与健康促进). 2011; 6(3):179-181.

S51	Shanghai	Yu YF, Liu JM, Liang KY, Tu CL, Chen Z, Chang W (余艳芳, 刘锦铭, 屠春林, 陈珠, 常伟). A community suvey of chronic abstructive pulmonary disease in Jiading District of Shanghai (上海市嘉定区社区慢性阻塞性肺疾病防治状况调查). Practical Geriatrics (实用老年医学). 2014; 28 (6): 478-483.
S52	Sichuan	Xia T, Huang XM (夏涛, 黄小明). Study on the Prevalence and risk factors of COPD in Yibin region (宜宾地区 COPD 患病情况及危险因素分析). China Practical Medicine (中国实用医药). 2010; 5(8): 251-252.
S53	Sichuan	Zeng XF, Wang XX, Bao Y, Chen F, Gao SS, Li K, Chen M, He H, Liu JK, Pu X, Du J (曾雪峰, 王晓霞, 包勇, 陈锋, 高蜀生, 李凯, 陈敏, 何慧, 刘健康, 蒲霞, 杜娟). Epidemiological survey on chronic obstructive pulmonary disease in Chengdu communitis. (成都市社区慢性阻塞性肺疾病流行病学调查). Chinese Journal of Respiratory and Critical Care Medicine (中国呼吸与危重监护杂志). 2011; 10(01): 30-32.
S54	Sichuan	Peng XP (彭小平). Prevalence rate and risk factors of chronic obstructive pulmonary disease in rural areas in Nanchong (农村居民慢性阻塞性肺疾病危险因素分析). Medicine Journal of West China (西部医学). 2011; 23(11): 2224-2227.
S55	Tianjin	Shan SX (单淑香). Basic data analysis for epidemiology investigation of chronic obstructive pulmonary disease (慢性阻塞性肺疾病流行病学调查基线资料分析). Postgraduate Thesis of Tianjin Medical University. 2005.
S56	Xinjiang	Xu ZX, Dong YL, Ai LT, Maimaitiabulaaishan, Kuerxijiang THT, Gulinner (徐忠星, 董宇莉, 艾来提, 买买提阿不拉艾山, 库尔西江·托乎提, 古丽努尔). A study of Chronic Obstructive Pulmonary Disease in the Taklimakan desert* (塔克拉玛干沙漠地区慢性阻塞性肺疾病调查研究). Chinese Journal of Prevention and Control of Chronic Non-Communicable Diseases (中国慢性病预防与控制). 2003;11(1)36.
S57	Xinjiang	Hu XT (胡晓婷). A survey of the prevalence rate and other related factors of chronic obstructive pulmonary disease of the older Uighur people in hetian, Xinjiang (新疆和田地区农村慢性阻塞性肺疾病的患病调查). Xinjiang Medical University Master's Thesis. 2010.
S58	Xinjiang	Ling M, Rong Y, Gou AQ, Niu Lm Wang H, Zulipeiya Aibaidula, Zhu J, Pang M, Ayiguli Abulaiti, Yu BQ, Wang HT, Zhao MH, Han KS (凌敏, 荣艳, 苟安栓, 牛灵, 王辉, 祖里培亚·艾拜都拉, 朱佳, 庞敏, 阿依古丽·阿布来提, 于碧磬, 汪海涛, 赵明华, 韩克斯). The risk factors for chronic obstructive pulmonary disease in Xinjiang rural areas (新疆农村地区慢性阻塞性肺疾病的危险因素调查). Chinese Journal of Tuberculosis and Respiratory Disease (中华结核和呼吸杂志). 2011; 34(9): 666-668.
S59	Yunan	Fan DM, Feng JL, Xu X, Ding HP, Sun YJ (樊冬梅, 封军莉, 徐兴, 丁华萍, 孙亚洁). Epidemic Situation and Influencing Factors of Chronic Diseases in Taixing (泰兴市慢性病流行情况及其影响因素). Occupation and Health (职业与健康). 2010; 26(3): 314-316.

S60	Yunan	Zhang X (张鑫). The epidemiological characteristic and risk factors of chronic obstructive Pulmonary disease (COPD) in Kunming city (昂明市居民慢性阻塞性肺疾病流行病学调查分析). Yunnan: Kunming Medical Univeristy (昆明医学院). 2011;1-71.
S61	Yunnan	Yao WQ, Dai BZ, He F, Wang M, Li Y, Yang XC, Zhao KW (姚为群, 戴百章, 何方, 王梅, 李玉, 杨陵程, 赵开武). A comparison of the COPD Prevalence between rural and urban residents* (城市居民与失地农民 COPD 患病率比较分析). Chinese Community Doctors (中国社区医师 (医学专业)). 2013;15(2):342-343.
S62	Zhejiang	Weng HX (翁海霞). An analysis of the relationship between smoking and the prevalence of COPD in the elderly in Wenzhou* (温州市老年人慢性阻塞性肺炎患病率与吸烟关系的调查). Starit Journal of Preventive Medicine (海峡预防医学杂志). 2005; 11(3): 75-76.
S63	Zhejiang	Wang YY, Li B, Wang L, Weng L, Li XX, Chen L, Ding J, Yu K, Chen ZB, Deng ZC (王雅艳,李冰,王龙,翁磊,李晓雯,陈磊,丁杰,俞凯,陈众博,邓在春). Prevalence survey on elderly patients with chronic obstructive pulmonary disease (老年人慢性阻塞性肺病流行状况调查). China Modern Doctor (中国现代医生). 2011; 49(35): 19-21.
S64	Zhejiang	Huang YH (黄月红). Survey of chronic obstructive pulmonary disease in Huzhou area (湖州地区慢性阻塞性肺疾病发病状况调查). China Modern Doctor (中国现代医生). 2012; 50(29):8-10.
S65	Zhejiang	Cui HL (崔宏林). An investigation into the Prevalence of COPD high-risk population* (慢性阻塞性肺疾病高危人群患病状况调查). China Modern Doctor (中国现代医生). 2012; 50(16): 12-14.
S66	Zhejiang	Chen ZB, Yu YM, Cao C, Ding QL, Lv D, Ma HY, Sun SF, Shu LH, Deng ZC (陈众博, 虞亦鸣, 曹超, 丁群力, 吕丹, 马红映, 孙士芳, 舒丽华, 邓在春). An investigation into the missing diagnosis status iof COPD among the elderly male residents in Ningbo Area* (宁波地区老年男性慢性阻塞性肺疾病漏诊情况调查研究). Chinese Journal of Rural Medicine and Pharmacy (中国乡村医药). 2013; 20(3): 60-61.
S67	Zhejiang	Xu X (徐鑫). An anaylsis on the risk factors among the smoking elderly residents in rural area* (农村中老年吸烟人群 COPD 发病危险因素分析). Practical Preventive Medicine (实用预防医学). 2013; 21(3): 376-378.
S68	Zhejiang	Weng L (翁磊).Analysis on epidemiological baseline data of COPD (慢性阻塞性肺疾病流行病学研究基线数据分析). Master's Thesis of Wenzhou Medical University. 2009.
S69	7 sites: Beijing, Tianjin, Liaoning	Zhou YM, Wang C, Yao WZ, Chen P, Kang J, Huang SG, Chen BY, Wang CZ, Ni DT, Wang XP, Wang DL, Liu SM, LU JC, Zheng JP, Zhong NS, Ran PX (周玉民, 王辰, 姚婉贞, 陈萍, 康健, 黄绍光, 陈宝元, 王长征, 倪殿涛, 王小平, 王大礼, 刘升明, 吕嘉春, 郑劲平, 钟南山, 冉丕鑫). Current status of prevention and management of chronic obstructive pulmonary

	Shanghai Guangdong Shanxi Chongqing	disease in rural area in China (中国农村慢性阻塞性肺疾病患病及防治现状). Chinese Journal of Internal Medicine (中华内科杂志). 2009;48(5):358-361.
S70	All 31 provinces	Yin P, Zhang M, Li Y, Jiang Y, Zhao W. Prevalence of COPD and its association with socioeconomic status in China: findings from China Chronic Disease Risk Factor Surveillance 2007. BMC Public Health. 201122;11:586. doi: 10.1186/1471-2458-11-586.
S71	5 urban areas in Harbin, Haikou, Liuzhou, Qingdao, Suzhou; 5 rural areas in Gansu, Henan, Hunan, Sichuan, Zhejiang	Kurmi, O. P., Li, L., Smith, M., Augustyn, M., Chen, J., Collins, R., et al. (2014). Regional variations in the prevalence and misdiagnosis of air flow obstruction in China: baseline results from a prospective cohort of the China Kadoorie Biobank (CKB). BMJ Open Respir Res, 1(1), e000025.

Table S2. Extracted data from the included studies on COPD prevalence in China

Study ID	Year	Gender	Setting	Mean age	Sample	Cases
S1	2003	Mixed	Rural	44	634	12
S1	2003	Mixed	Rural	54.5	428	20
S1	2003	Mixed	Rural	64.5	319	44
S1	2003	Mixed	Rural	74.5	181	51
S1	2003	Mixed	Rural	85.5	62	21
S2	2007	Male	Mixed	62.5	394	102
S2	2007	Female	Mixed	62.5	206	15
S3	2009	Mixed	Urban	44.5	210	16
S3	2009	Mixed	Urban	54.5	442	43
S3	2009	Mixed	Urban	64.5	274	47
S3	2009	Mixed	Urban	74.5	271	81
S3	2009	Mixed	Urban	85	56	25
S4	2008	Male	Urban	59.8	382	39
S4	2008	Female	Urban	57.1	879	38
S5	1991.5	Male	Rural	19.5	3358	6
S5	1991.5	Male	Rural	29.5	3358	34
S5	1991.5	Male	Rural	39.5	2938	94
S5	1991.5	Male	Rural	49.5	1666	149
S5	1991.5	Male	Rural	59.5	1414	239
S5	1991.5	Male	Rural	69.5	910	146
S5	1991.5	Male	Rural	80	335	30
S5	1991.5	Female	Rural	19.5	3146	2
S5	1991.5	Female	Rural	29.5	3584	11
S5	1991.5	Female	Rural	39.5	3772	43
S5	1991.5	Female	Rural	49.5	2269	81
S5	1991.5	Female	Rural	59.5	1347	86
S5	1991.5	Female	Rural	69.5	1049	83
S5	1991.5	Female	Rural	80	470	12
S6	2003	Male	Urban	44.5	142	8
S6	2003	Male	Urban	54.5	117	15
S6	2003	Male	Urban	64.5	97	34
S6	2003	Male	Urban	77.5	161	62
S6	2003	Female	Urban	44.5	213	6
S6	2003	Female	Urban	54.5	325	14
S6	2003	Female	Urban	64.5	203	18
S6	2003	Female	Urban	77.5	260	37
S7	2007	Mixed	Urban	44.5	347	6
S7	2007	Mixed	Urban	54.5	822	36
S7	2007	Mixed	Urban	64.5	415	46
S7	2007	Mixed	Urban	77.5	440	72
S8	2001	Male	Urban	44.5	78	2

S8	2001	Male	Urban	54.5	57	5
S8	2001	Male	Urban	64.5	106	14
S8	2001	Male	Urban	77.5	87	21
S8	2001	Female	Urban	44.5	167	3
S8	2001	Female	Urban	54.5	116	1
S8	2001	Female	Urban	64.5	173	7
S8	2001	Female	Urban	77.5	110	14
S9	2002.5	Male	Urban	44.5	185	6
S9	2002.5	Male	Urban	54.5	136	13
S9	2002.5	Male	Urban	64.5	215	35
S9	2002.5	Male	Urban	77.5	194	47
S9	2002.5	Male	Rural	44.5	220	16
S9	2002.5	Male	Rural	54.5	160	21
S9	2002.5	Male	Rural	64.5	179	48
S9	2002.5	Male	Rural	77.5	81	32
S9	2002.5	Female	Urban	44.5	344	3
S9	2002.5	Female	Urban	54.5	245	1
S9	2002.5	Female	Urban	64.5	310	8
S9	2002.5	Female	Urban	77.5	189	21
S9	2002.5	Female	Rural	44.5	380	16
S9	2002.5	Female	Rural	54.5	212	10
S9	2002.5	Female	Rural	64.5	165	22
S9	2002.5	Female	Rural	77.5	71	11
S10	2005	Male	Mixed	44.5	257	18
S10	2005	Male	Mixed	54.5	225	21
S10	2005	Male	Mixed	64.5	145	30
S10	2005	Male	Mixed	77.5	153	38
S10	2005	Female	Mixed	44.5	98	2
S10	2005	Female	Mixed	54.5	88	3
S10	2005	Female	Mixed	64.5	75	12
S10	2005	Female	Mixed	77.5	59	14
S11	2003.5	Mixed	Urban	44.5	245	0
S11	2003.5	Mixed	Urban	54.5	295	6
S11	2003.5	Mixed	Urban	64.5	261	10
S11	2003.5	Mixed	Urban	77.5	325	37
S11	2003.5	Male	Rural	44.5	102	1
S11	2003.5	Male	Rural	54.5	131	9
S11	2003.5	Male	Rural	64.5	125	17
S11	2003.5	Male	Rural	77.5	165	31
S11	2003.5	Female	Rural	44.5	225	0
S11	2003.5	Female	Rural	54.5	206	4
S11	2003.5	Female	Rural	64.5	168	11
S11	2003.5	Female	Rural	77.5	187	19

S12	2009	Male	Rural	44.5	95	3
S12	2009	Male	Rural	54.5	184	18
S12	2009	Male	Rural	64.5	156	22
S12	2009	Male	Rural	74.5	82	26
S12	2009	Male	Rural	83.5	3	2
S12	2009	Female	Rural	44.5	65	1
S12	2009	Female	Rural	54.5	144	5
S12	2009	Female	Rural	64.5	209	13
S12	2009	Female	Rural	74.5	75	10
S12	2009	Female	Rural	83.5	6	4
S13	2008	Male	Mixed	66.5	452	84
S13	2008	Female	Mixed	66.5	498	92
S14	2002	Male	Urban	44.5	106	4
S14	2002	Male	Urban	54.5	60	6
S14	2002	Male	Urban	64.5	43	15
S14	2002	Male	Urban	77.5	27	11
S14	2002	Female	Urban	44.5	98	4
S14	2002	Female	Urban	54.5	65	1
S14	2002	Female	Urban	64.5	54	7
S14	2002	Female	Urban	77.5	20	6
S15	2011	Female	Rural	45	149	7
S16	2008	Male	Rural	44.5	331	17
S16	2008	Male	Rural	54.5	235	25
S16	2008	Male	Rural	64.5	292	43
S16	2008	Male	Rural	77	152	49
S16	2008	Female	Rural	44.5	368	13
S16	2008	Female	Rural	54.5	214	10
S16	2008	Female	Rural	64.5	187	24
S16	2008	Female	Rural	77	169	28
S17	2008	Male	Mixed	57	896	72
S17	2008	Female	Mixed	57	827	63
S18	2009	Male	Rural	45	233	13
S18	2009	Male	Rural	55.5	165	15
S18	2009	Male	Rural	65.5	140	27
S18	2009	Male	Rural	78	128	33
S18	2009	Female	Rural	45	287	14
S18	2009	Female	Rural	55.5	252	28
S18	2009	Female	Rural	65.5	181	16
S18	2009	Female	Rural	78	123	24
S19	2010	Male	Urban	44.5	152	6
S19	2010	Male	Urban	54.5	129	6
S19	2010	Male	Urban	64.5	133	7
S19	2010	Male	Urban	77.5	92	1

S19	2010	Female	Urban	44.5	166	1
S19	2010	Female	Urban	54.5	188	5
S19	2010	Female	Urban	64.5	135	4
S19	2010	Female	Urban	77.5	89	2
S20	2006	Male	Rural	44.5	286	16
S20	2006	Male	Rural	54.5	263	30
S20	2006	Male	Rural	64.5	241	48
S20	2006	Male	Rural	77.5	102	28
S20	2006	Female	Rural	44.5	352	9
S20	2006	Female	Rural	54.5	296	12
S20	2006	Female	Rural	64.5	225	23
S20	2006	Female	Rural	77.5	118	20
S21	2009	Mixed	Rural	44.5	217	3
S21	2009	Mixed	Rural	54.5	466	18
S21	2009	Mixed	Rural	64.5	492	68
S21	2009	Mixed	Rural	77.5	379	84
S22	2006	Mixed	Urban	19.5	571	1
S22	2006	Mixed	Urban	29.5	437	3
S22	2006	Mixed	Urban	39.5	1034	19
S22	2006	Mixed	Urban	49.5	922	40
S22	2006	Mixed	Urban	59.5	657	55
S22	2006	Mixed	Urban	75	619	86
S22	2006	Mixed	Rural	19.5	765	0
S22	2006	Mixed	Rural	29.5	671	2
S22	2006	Mixed	Rural	39.5	773	16
S22	2006	Mixed	Rural	49.5	784	43
S22	2006	Mixed	Rural	59.5	536	67
S22	2006	Mixed	Rural	75	474	85
S23	2008	Male	Urban	44.5	136	4
S23	2008	Male	Urban	54.5	124	7
S23	2008	Male	Urban	64.5	104	18
S23	2008	Male	Urban	77.5	156	37
S23	2008	Female	Urban	44.5	115	2
S23	2008	Female	Urban	54.5	121	3
S23	2008	Female	Urban	64.5	93	7
S23	2008	Female	Urban	77.5	161	13
S24	2007	Mixed	Rural	44.5	892	62
S24	2007	Mixed	Rural	54.5	1023	95
S24	2007	Mixed	Rural	64.5	754	121
S24	2007	Mixed	Rural	77.5	585	125
S25	2003	Male	Rural	44.5	334	5
S25	2003	Male	Rural	54.5	301	13
S25	2003	Male	Rural	64.5	200	28

S25	2003	Male	Rural	77.5	97	28
S25	2003	Female	Rural	44.5	411	6
S25	2003	Female	Rural	54.5	404	24
S25	2003	Female	Rural	64.5	172	20
S25	2003	Female	Rural	77.5	91	14
S26	2003	Mixed	Urban	44.5	563	10
S26	2003	Mixed	Urban	54.5	408	23
S26	2003	Mixed	Urban	64.5	627	64
S26	2003	Mixed	Urban	74.5	329	58
S26	2003	Mixed	Urban	83.5	30	2
S27	2007	Male	Rural	25.5	212	0
S27	2007	Male	Rural	34.5	638	0
S27	2007	Male	Rural	44.5	851	113
S27	2007	Male	Rural	54.5	771	243
S27	2007	Male	Rural	67	188	125
S27	2007	Female	Rural	25.5	220	2
S27	2007	Female	Rural	34.5	662	5
S27	2007	Female	Rural	44.5	883	101
S27	2007	Female	Rural	54.5	800	257
S27	2007	Female	Rural	67	195	101
S28	2008	Male	Urban	37	99	0
S28	2008	Male	Urban	44.5	174	3
S28	2008	Male	Urban	54.5	320	11
S28	2008	Male	Urban	64.5	172	10
S28	2008	Male	Urban	75	124	20
S28	2008	Female	Urban	37	103	0
S28	2008	Female	Urban	44.5	268	5
S28	2008	Female	Urban	54.5	492	12
S28	2008	Female	Urban	64.5	213	14
S28	2008	Female	Urban	75	236	37
S29	2000	Male	Urban	60	736	96
S29	2000	Female	Urban	60	756	53
S31	2005	Male	Mixed	7	702	8
S31	2005	Male	Mixed	29.5	2566	90
S31	2005	Male	Mixed	52	927	130
S31	2005	Male	Mixed	72.5	688	164
S31	2005	Female	Mixed	7	560	3
S31	2005	Female	Mixed	29.5	2601	43
S31	2005	Female	Mixed	52	915	68
S31	2005	Female	Mixed	72.5	817	143
S32	2004	Mixed	Rural	44.5	1592	107
S32	2004	Mixed	Rural	54.5	1749	185
S32	2004	Mixed	Rural	64.5	1128	237

S32	2004	Mixed	Rural	77.5	1034	278
S33	2003	Male	Rural	44.5	70	0
S33	2003	Male	Rural	54.5	64	4
S33	2003	Male	Rural	64.5	42	6
S33	2003	Male	Rural	77.5	20	6
S33	2003	Female	Rural	44.5	82	2
S33	2003	Female	Rural	54.5	80	4
S33	2003	Female	Rural	64.5	34	3
S33	2003	Female	Rural	77.5	18	3
S35	2009	Male	Rural	72.5	816	180
S35	2009	Female	Rural	72.5	1008	101
S36	2009	Male	Urban	44.5	314	18
S36	2009	Male	Urban	54.5	231	23
S36	2009	Male	Urban	64.5	244	39
S36	2009	Male	Urban	77.5	123	31
S36	2009	Female	Urban	44.5	437	4
S36	2009	Female	Urban	54.5	304	9
S36	2009	Female	Urban	64.5	312	22
S36	2009	Female	Urban	77.5	90	10
S37	2003	Male	Rural	49.5	995	52
S37	2003	Male	Rural	78	816	180
S37	2003	Female	Rural	49.5	1228	14
S37	2003	Female	Rural	78	1008	101
S38	2011	Mixed	Rural	29	966	67
S38	2011	Mixed	Rural	45.5	1252	96
S38	2011	Mixed	Rural	55.5	754	126
S38	2011	Mixed	Rural	68	596	133
S39	2010	Male	Rural	44.5	174	17
S39	2010	Male	Rural	54.5	182	23
S39	2010	Male	Rural	64.5	186	30
S39	2010	Male	Rural	77.5	150	29
S39	2010	Female	Rural	44.5	183	8
S39	2010	Female	Rural	54.5	187	11
S39	2010	Female	Rural	64.5	185	19
S39	2010	Female	Rural	77.5	162	22
S40	1987.5	Mixed	Rural	44	1689	121
S41	1988.5	Male	Rural	44	2673	260
S41	1988.5	Female	Rural	44	2893	154
S43	2002	Male	Urban	72.5	255	33
S43	2002	Male	Rural	72.5	294	71
S43	2002	Female	Urban	72.5	384	18
S43	2002	Female	Rural	72.5	281	25
S44	2003	Mixed	Urban	62.5	624	65

S45	2005	Male	Urban	58	1710	80
S45	2005	Female	Urban	58	1406	33
S46	2008	Male	Urban	44.5	76	6
S46	2008	Male	Urban	54.5	172	11
S46	2008	Male	Urban	64.5	72	8
S46	2008	Male	Urban	74.5	92	26
S46	2008	Male	Urban	86	29	13
S46	2008	Female	Urban	44.5	56	1
S46	2008	Female	Urban	54.5	194	8
S46	2008	Female	Urban	64.5	90	6
S46	2008	Female	Urban	74.5	105	15
S46	2008	Female	Urban	86	40	8
S47	2008	Mixed	Urban	64.5	408	32
S47	2008	Mixed	Urban	74.5	467	63
S47	2008	Mixed	Urban	82.5	168	30
S48	2009	Male	Urban	62	1312	97
S48	2009	Female	Urban	62	1388	44
S49	2007	Mixed	Urban	44.5	178	10
S49	2007	Mixed	Urban	54.5	380	4
S49	2007	Mixed	Urban	64.5	334	32
S49	2007	Mixed	Urban	74.5	360	62
S49	2007	Mixed	Urban	82.5	78	24
S50	2010	Male	Urban	44.5	246	10
S50	2010	Male	Urban	54.5	608	36
S50	2010	Male	Urban	64.5	372	28
S50	2010	Male	Urban	74.5	438	32
S50	2010	Female	Urban	44.5	192	0
S50	2010	Female	Urban	54.5	426	2
S50	2010	Female	Urban	64.5	268	6
S50	2010	Female	Urban	74.5	340	12
S51	2011	Mixed	Urban	44.5	40	0
S51	2011	Mixed	Urban	54.5	144	10
S51	2011	Mixed	Urban	64.5	1030	74
S51	2011	Mixed	Urban	74.5	694	57
S51	2011	Mixed	Urban	84.5	100	24
S52	2007	Mixed	Urban	44.5	103	8
S52	2007	Mixed	Urban	54.5	95	16
S52	2007	Mixed	Urban	64.5	91	21
S52	2007	Mixed	Urban	77.5	65	21
S52	2007	Mixed	Rural	44.5	187	9
S52	2007	Mixed	Rural	54.5	164	17
S52	2007	Mixed	Rural	64.5	151	24
S52	2007	Mixed	Rural	77.5	118	25

S53	2008	Male	Urban	44.5	341	14
S53	2008	Male	Urban	54.5	373	30
S53	2008	Male	Urban	64.5	423	71
S53	2008	Male	Urban	77.5	402	101
S53	2008	Female	Urban	44.5	615	15
S53	2008	Female	Urban	54.5	514	23
S53	2008	Female	Urban	64.5	602	45
S53	2008	Female	Urban	77.5	417	55
S54	2008	Male	Rural	19.5	84	5
S54	2008	Male	Rural	49.5	290	19
S54	2008	Male	Rural	64.5	600	72
S54	2008	Male	Rural	75	130	58
S54	2008	Female	Rural	19.5	33	1
S54	2008	Female	Rural	49.5	158	7
S54	2008	Female	Rural	64.5	360	21
S54	2008	Female	Rural	75	129	27
S55	2003	Male	Rural	44.5	195	6
S55	2003	Male	Rural	54.5	211	28
S55	2003	Male	Rural	64.5	160	28
S55	2003	Male	Rural	74.5	92	25
S55	2003	Male	Rural	82.5	9	3
S55	2003	Female	Rural	44.5	391	9
S55	2003	Female	Rural	54.5	245	7
S55	2003	Female	Rural	64.5	129	17
S55	2003	Female	Rural	74.5	65	15
S55	2003	Female	Rural	82.5	11	4
S55	2003	Mixed	Urban	44.5	340	8
S55	2003	Mixed	Urban	54.5	356	15
S55	2003	Mixed	Urban	64.5	441	52
S55	2003	Mixed	Urban	74.5	319	61
S55	2003	Mixed	Urban	82.5	44	12
S56	2000.5	Male	Mixed	29.5	462	16
S56	2000.5	Male	Mixed	39.5	589	30
S56	2000.5	Male	Mixed	49.5	890	52
S56	2000.5	Male	Mixed	59.5	1004	78
S56	2000.5	Male	Mixed	69.5	1061	99
S56	2000.5	Male	Mixed	80	1069	120
S56	2000.5	Female	Mixed	29.5	450	14
S56	2000.5	Female	Mixed	39.5	573	29
S56	2000.5	Female	Mixed	49.5	891	54
S56	2000.5	Female	Mixed	59.5	999	79
S56	2000.5	Female	Mixed	69.5	1048	104
S56	2000.5	Female	Mixed	80	971	105

S57	2009	Male	Rural	64.5	56	8
S57	2009	Male	Rural	74.5	77	12
S57	2009	Male	Rural	84.5	56	12
S57	2009	Male	Rural	102.5	22	5
S57	2009	Female	Rural	64.5	42	13
S57	2009	Female	Rural	74.5	48	12
S57	2009	Female	Rural	84.5	31	9
S57	2009	Female	Rural	102.5	10	0
S58	2008.5	Mixed	Rural	22	1110	9
S58	2008.5	Mixed	Rural	39.5	1606	35
S58	2008.5	Mixed	Rural	59.5	643	73
S58	2008.5	Mixed	Rural	81	130	21
S59	2007	Mixed	Mixed	70	5510	166
S60	2010	Mixed	Urban	44.5	720	1
S60	2010	Mixed	Urban	54.5	588	4
S60	2010	Mixed	Urban	64.5	505	30
S60	2010	Mixed	Urban	77.5	380	57
S61	2010	Mixed	Urban	50.44	78	4
S61	2010	Mixed	Rural	50.77	94	8
S62	2002	Male	Mixed	64.5	399	33
S62	2002	Male	Mixed	74.5	177	21
S62	2002	Male	Mixed	82.5	29	5
S62	2002	Female	Mixed	64.5	382	8
S62	2002	Female	Mixed	74.5	191	10
S62	2002	Female	Mixed	82.5	31	2
S63	2010	Male	Urban	76.47	363	69
S63	2010	Male	Rural	79.12	394	64
S63	2010	Female	Urban	76.47	359	40
S63	2010	Female	Rural	79.12	351	66
S64	2010.5	Male	Mixed	44.5	350	18
S64	2010.5	Male	Mixed	54.5	257	28
S64	2010.5	Male	Mixed	64.5	339	71
S64	2010.5	Male	Mixed	77.5	236	66
S64	2010.5	Female	Mixed	44.5	626	14
S64	2010.5	Female	Mixed	54.5	393	15
S64	2010.5	Female	Mixed	64.5	410	29
S64	2010.5	Female	Mixed	77.5	225	30
S65	2009	Male	Mixed	44.5	157	39
S65	2009	Male	Mixed	54.5	254	42
S65	2009	Male	Mixed	64.5	298	63
S65	2009	Male	Mixed	77.5	322	83
S65	2009	Female	Mixed	44.5	109	18
S65	2009	Female	Mixed	54.5	185	27

S65	2009	Female	Mixed	64.5	283	32
S65	2009	Female	Mixed	77.5	319	38
S66	2009	Male	Mixed	65	509	74
S66	2009	Male	Mixed	78	806	118
S67	2011	Mixed	Rural	49.5	606	49
S67	2011	Mixed	Rural	70.5	492	76
S68	2008.5	Male	Mixed	44.5	166	9
S68	2008.5	Male	Mixed	54.5	168	18
S68	2008.5	Male	Mixed	64.5	82	16
S68	2008.5	Male	Mixed	74.5	56	16
S68	2008.5	Male	Mixed	84.5	37	13
S68	2008.5	Female	Mixed	44.5	209	6
S68	2008.5	Female	Mixed	54.5	214	7
S68	2008.5	Female	Mixed	64.5	103	6
S68	2008.5	Female	Mixed	74.5	71	7
S68	2008.5	Female	Mixed	84.5	46	5
S69	2003	Mixed	Urban	44.5	3280	59
S69	2003	Mixed	Urban	54.5	2554	102
S69	2003	Mixed	Urban	64.5	2637	264
S69	2003	Mixed	Urban	77.5	2340	413
S69	2003	Male	Rural	44.5	1385	51
S69	2003	Male	Rural	54.5	1311	111
S69	2003	Male	Rural	64.5	1017	188
S69	2003	Male	Rural	77.5	610	203
S69	2003	Female	Rural	44.5	2077	43
S69	2003	Female	Rural	54.5	1652	65
S69	2003	Female	Rural	64.5	864	78
S69	2003	Female	Rural	77.5	518	91
S70	2007	Mixed	Urban	43.50467	19328	484
S70	2007	Mixed	Rural	43.50467	30035	939
S71	2006	Male	Urban	53.1	91220	4105
S71	2006	Male	Rural	52.6	118837	9982
S71	2006	Female	Urban	52.6	134711	4445
S71	2006	Female	Rural	50.5	167727	9225
